# Supplementary material for: A Modified 1H-NMR Quantification Method of Ephedrine Alkaloids in Ephedrae Herba Samples
Source: Int J Mol Sci. 2023 Jul 10;24(14):11272. doi: 10.3390/ijms241411272 (PMC10378770; doi:10.3390/ijms241411272)
Supplement: Supplementary file 1 [file ijms-24-11272-s001.zip › ijms-2504927-supplementary.pdf]

Supplementary Materials

A Modified ^{1}H-NMR Quantification Method of Ephedrine Alkaloids in Ephedrae Herba Samples

Yue-Chiun Li [1,†], Chia-Hung Wu [2,†], Thi Ha Le [1], Qingjun Yuan [3], Luqi Huang [3], Guo-Fen Chen [4], Mei-Lin Yang [1], Sio-Hong Lam [1], Hsin-Yi Hung [1], Handong Sun [5], Yi-Hung Wu [6], Ping-Chung Kuo [1,*] and Tian-Shung Wu [1,*]

1 School of Pharmacy, College of Medicine, National Cheng Kung University, Tainan 701, Taiwan; ycli0126@gmail.com (Y.-C.L.); lethiha.hup@gmail.com (T.H.L.); l3891104@nckualumni.org.tw (M.-L.Y.); shlam@mail.ncku.edu.tw (S.-H.L.); z10308005@email.ncku.edu.tw (H.-Y.H.)

2 School of Post-Baccalaureate Chinese Medicine, China Medical University, Taichung 404, Taiwan; 108030049@365.cmu.edu.tw

3 State Key Laboratory Breeding Base of Dao-di Herbs, National Resource Center for Chinese Materia Medica, China Academy of Chinese Medical Sciences, Beijing 100010, China; yuanqingjun@icmm.ac.cn (Q.Y.); huangluqi01@126.com (L.H.)

4 Department of Chemistry, National Chung-Hsing University, Taichung 402, Taiwan; edward.chen@jeolanalytical.com.tw

5 Kunming Institute of Botany, Chinese Academy of Sciences, Kunming 650201, China; hdsun@mail.kib.ac.cn

6 Hsinhua Forest Station, The Experimental Forest Management Office, National Chung-Hsing University, Taichung 402, Taiwan; yihung@dragon.nchu.edu.tw

* Correspondence: z10502016@ncku.edu.tw (P.-C.K.); tswu@mail.ncku.edu.tw (T.-S.W.); Tel.: +886-6-2353535 (ext. 6806) (P.-C.K.); +886-6-2757575 (ext. 65333) (T.-S.W.)

† These authors contributed equally to this work.

Contents

Figure S1. The ^{1}H-NMR spectra of ephedrine alkaloids extract of EP01, EP02, and EP03.

Figure S2. Ephedrine alkaloids and their cyclization products in EP01, EP02, and EP03.

Figure S3. ESI-TOF-MS of compound **7**.

Figure S4. ^{1}H-NMR of compound **7**.

Figure S5. Characteritic carbon signals of compound **7**.

Figure S6. HMBC of compound **7**.

Figure S7. ESI-TOF-MS of compound **8**.

Figure S8. ^{1}H-NMR of compound **8**.

Figure S9. Characteritic carbon signals of compound **8**.

Figure S10. HMBC of compound **8**.

Figure S11. ^{1}H-NMR of compound **9**.

Figure S12. Characteritic carbon signals of compound **9**.

Figure S13. HMBC of compound **9**.

Figure S14. The ephedrine alkaloids chloroform extracts of *E. sinica* (EP01).

Figure S15. The ephedrine alkaloids benzene extracts of *E. sinica* (EP04).

Figure S16. ^{1}H-NMR of ME (**1**).

Figure S17. HSQC/TOCSY (——), HSQC (.....) and HMBC (— —) spectra of ME (**1**).

Figure S18. ^{1}H-NMR of EP (**2**).

Figure S19. HSQC/TOCSY (——), HSQC (.....) and HMBC(— —) spectra of EP (**2**).

Figure S20. ^{1}H-NMR of NP (**4**).

Figure S21. HSQC/TOCSY (——) spectra of NP (**4**).

Figure S22. ^{1}H-NMR of PE (**6**).

Figure S23. HSQC/TOCSY (——), HSQC (.....) and HMBC(— —) spectra of PE (**6**).

Figure S24. ^{1}H-NMR of EP01.

Figure S25. ^{1}H-NMR of EP02.

Figure S26. ^{1}H-NMR of EP03.

Figure S27. ^{1}H-NMR of EP04.

Figure S28. ^{1}H-NMR of EP05.

Figure S29. ^{1}H-NMR of EP06.

Figure S30. ^{1}H-NMR of EP07.

Figure S31. ^{1}H-NMR of EP08.

Figure S32. ^{1}H-NMR of EP09.
Figure S33. ^{1}H-NMR of EP10.
Figure S34. ^{1}H-NMR of EP11.
Figure S35. ^{1}H-NMR of EP12.
Figure S36. ^{1}H-NMR of EP13.
Figure S37. ^{1}H-NMR of EP14.
Figure S38. ^{1}H-NMR of EP15.
Figure S39. ^{1}H-NMR of EP16.
Figure S40. ^{1}H-NMR of EP17.
Figure S41. ^{1}H-NMR of EP18.
Figure S42. ^{1}H-NMR of EP19.
Figure S43. ^{1}H-NMR of EP20.
Table S1. Extraction and preparation of Ephedrae Herba for NMR analysis.

Figure S1. The ^{1}H-NMR spectra of ephedrine alkaloids extract of EP01, EP02, and EP03.

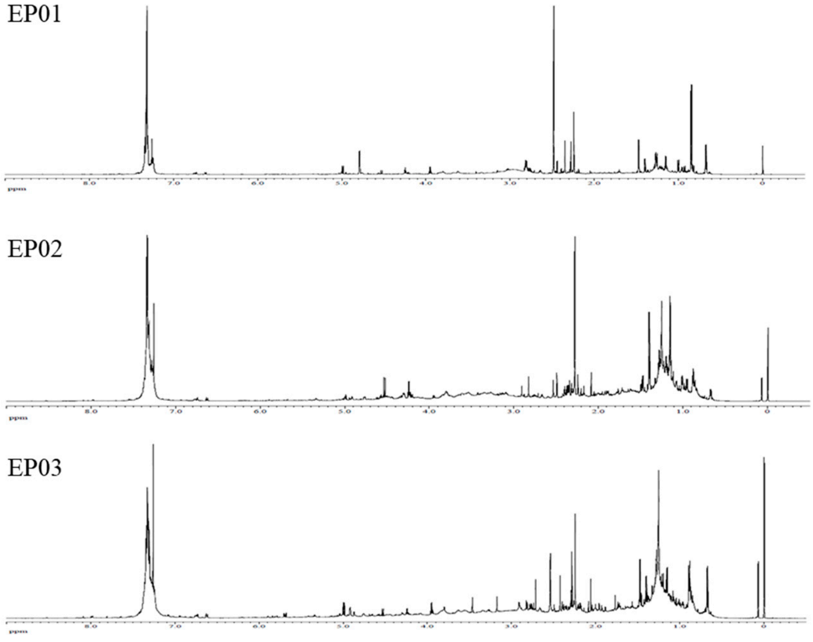

Figure S2. Ephedrine alkaloids and their cyclization products in EP01, EP02, and EP03.

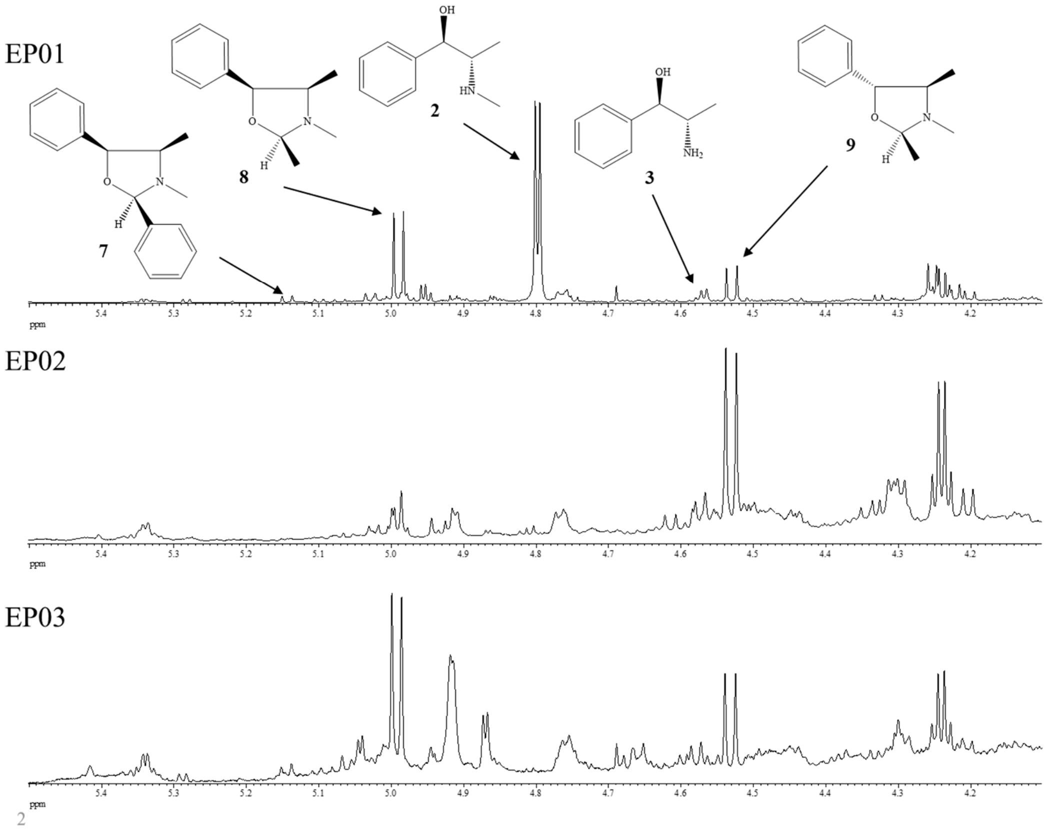

Figure S3. ESI-TOF-MS of compound **7**.

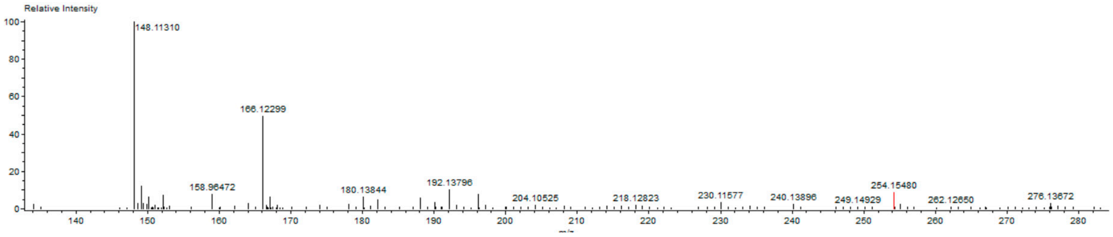

Mass	Intensity	Calc. Mass	Mass Difference [mDa]	Mass Difference [ppm]	Possible Formula	Unsaturation Number
254.15480	17467.03	254.15449	0.31	1.23	12C17 1H20 14N 16O	8.5

Figure S4. ^{1}H-NMR of compound **7**.

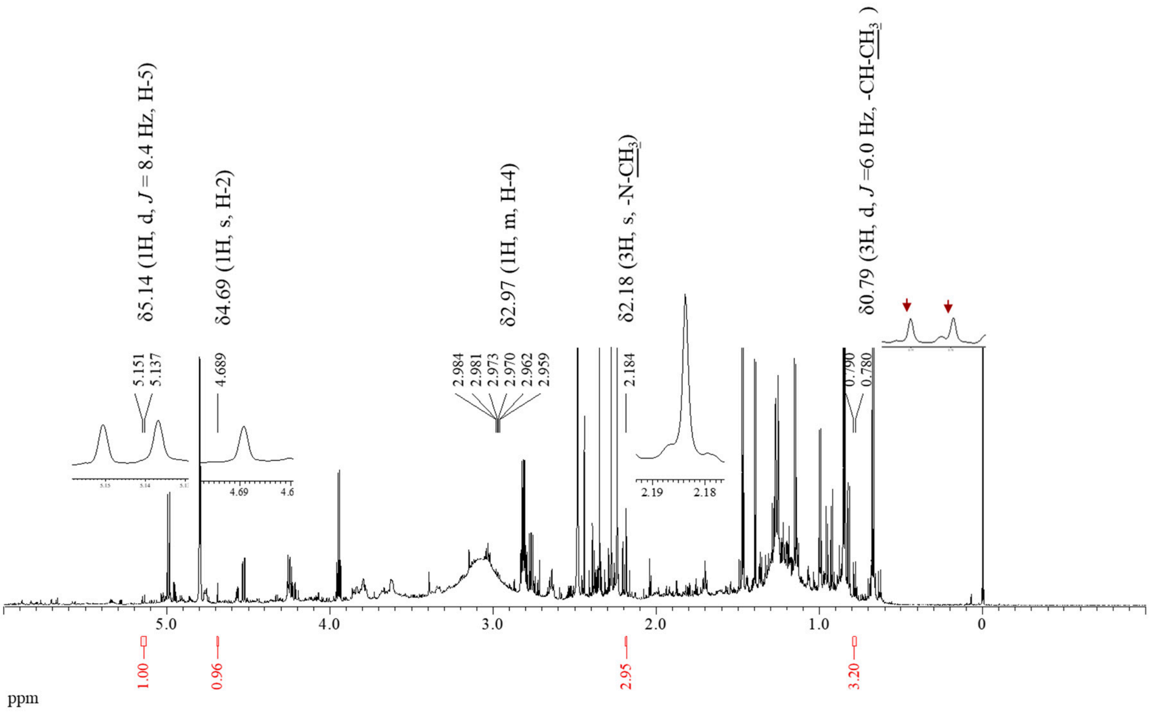

Figure S5. Characteritic carbon signals of compound **7**.

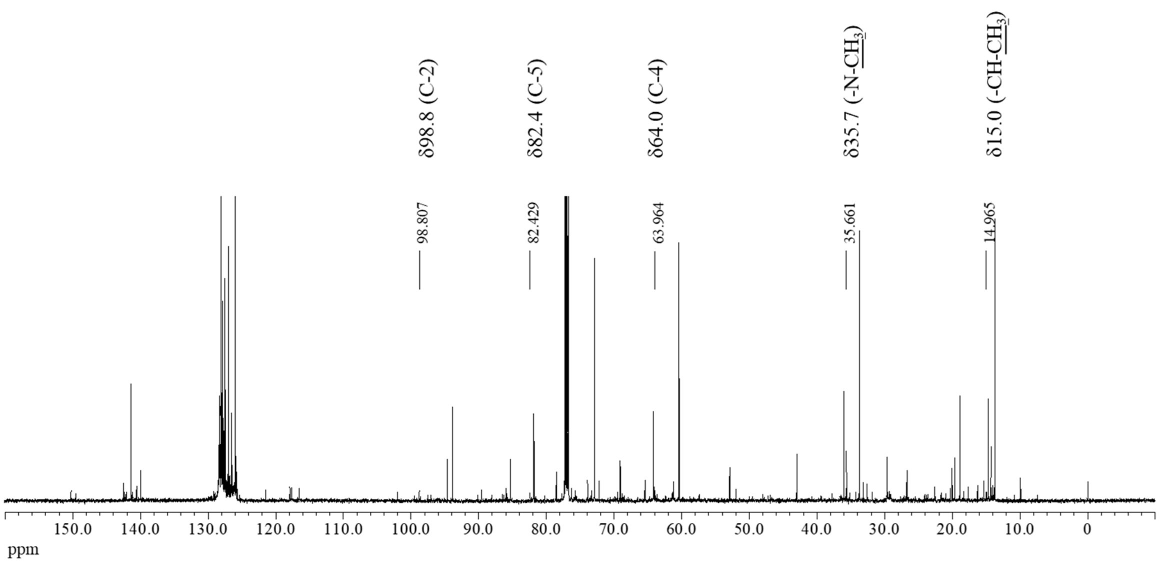

Figure S6. HMBC of compound **7**.

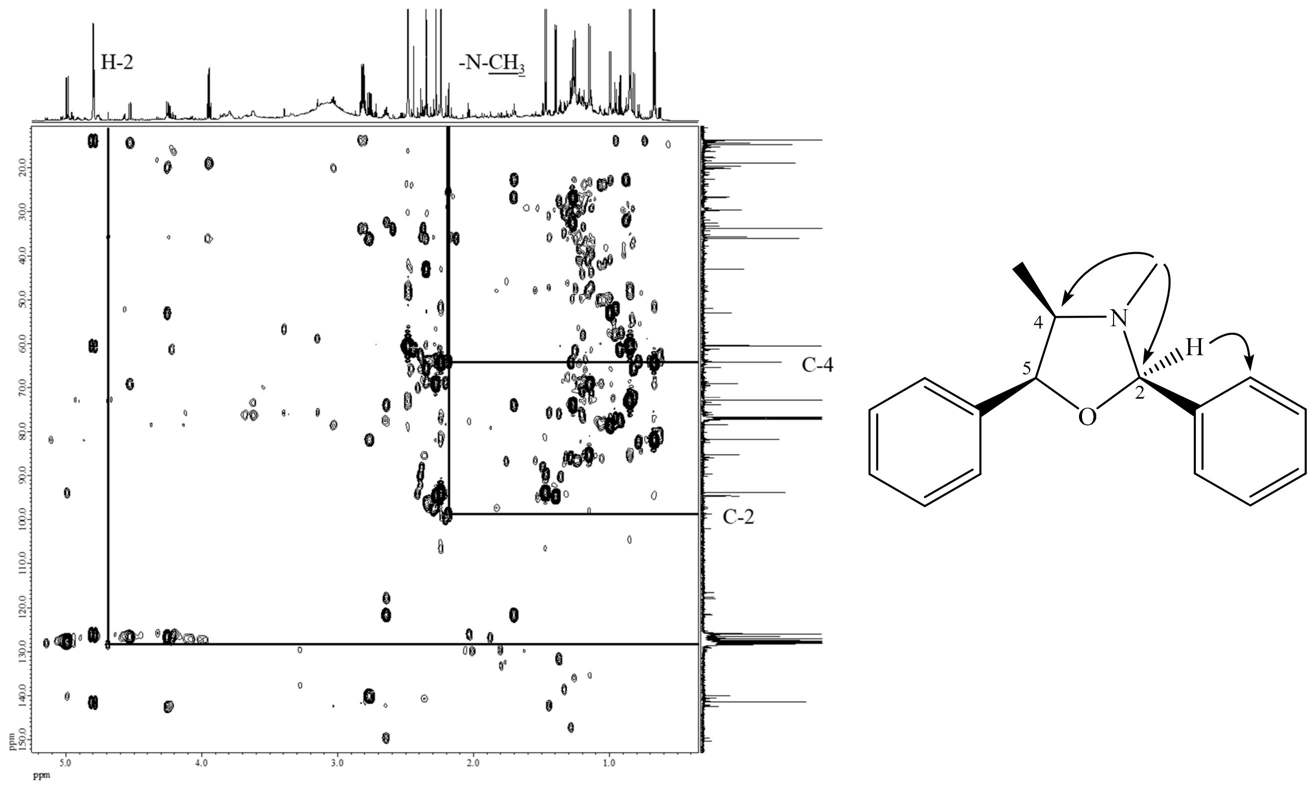

Figure S7. ESI-TOF-MS of compound **8**.

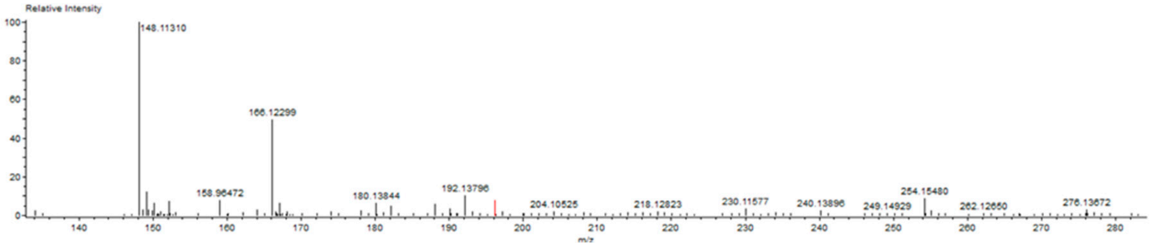

Mass	Intensity	Calc. Mass	Mass Difference [mDa]	Mass Difference [ppm]	Possible Formula	Unsaturation Number
192.13796	20013.41	192.13884	-0.88	-4.55	12C12 1H18 14N 16O	4.5

Figure S8. ^{1}H-NMR of compound **8**.

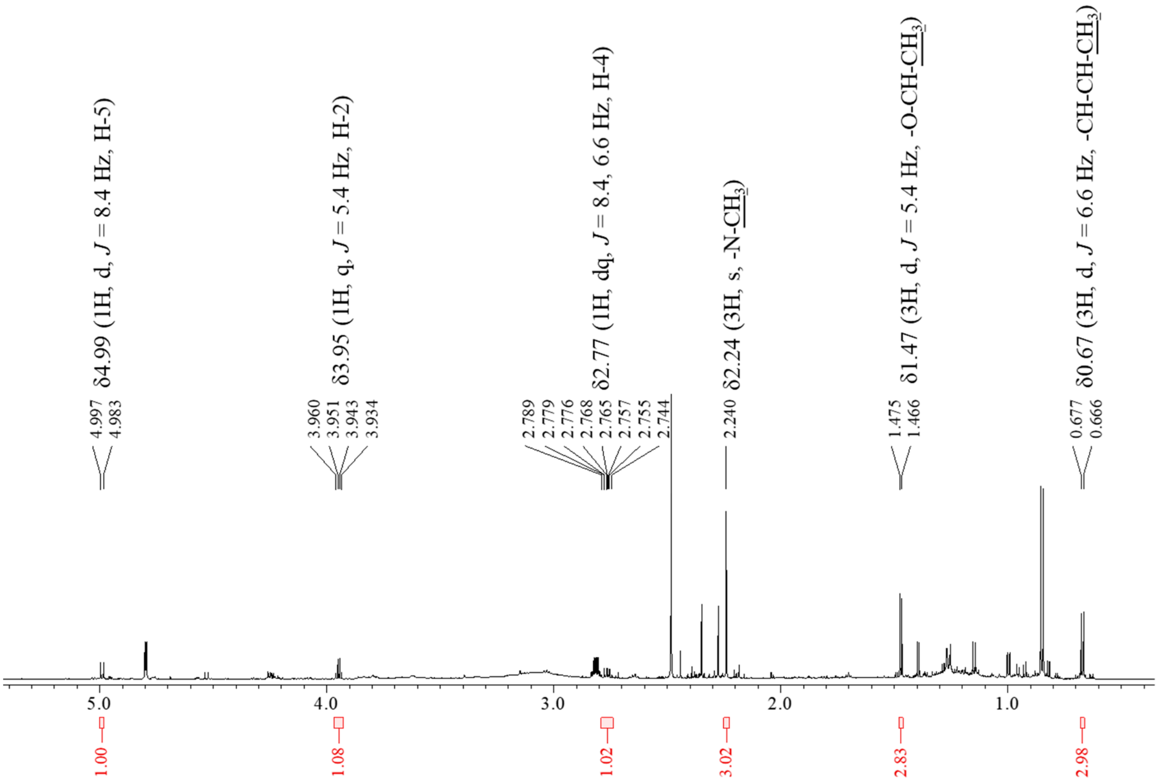

Figure S9. Characteritic carbon signals of compound **8**.

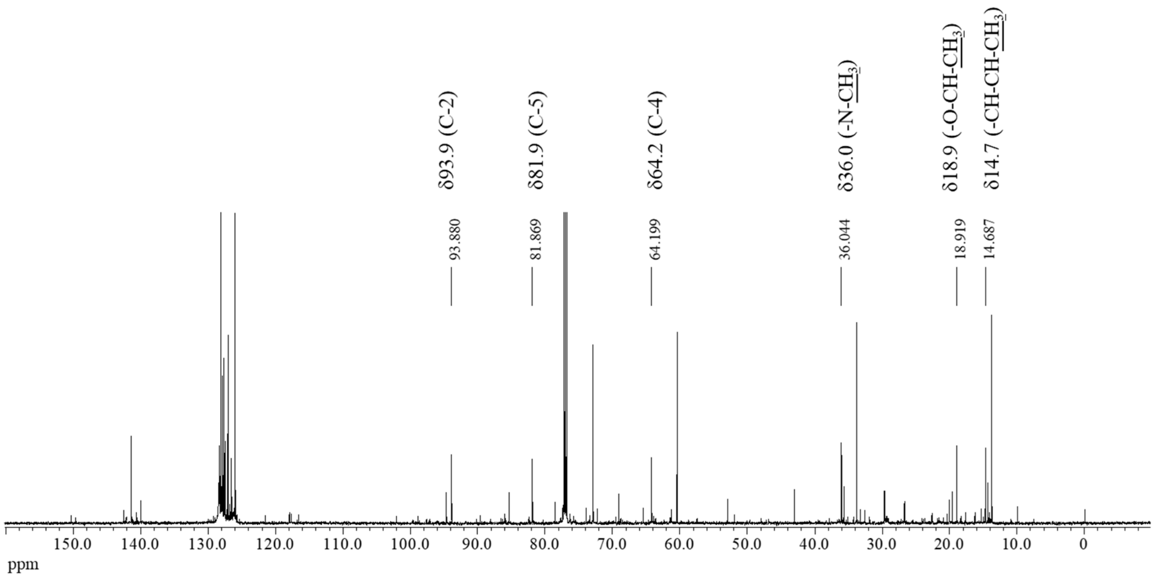

Figure S10. HMBC of compound **8**.

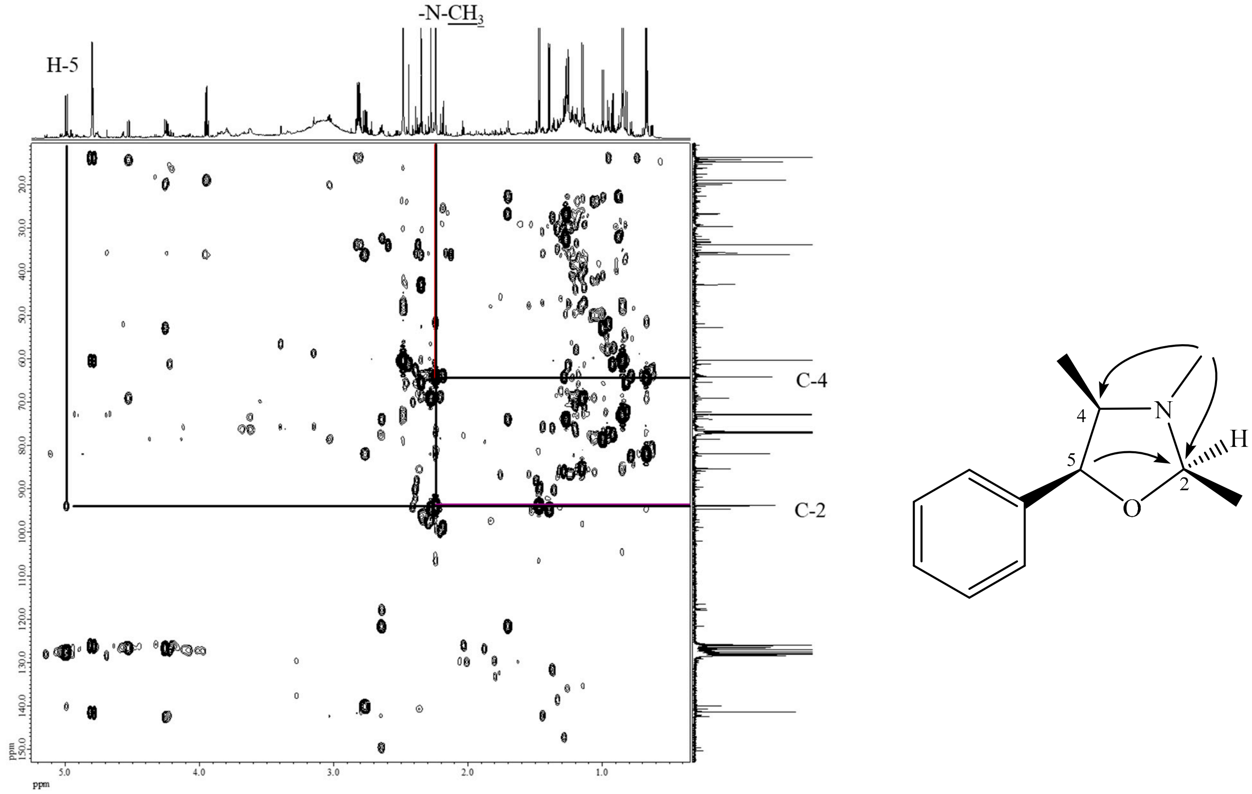

Figure S11. ^{1}H-NMR of compound **9**.

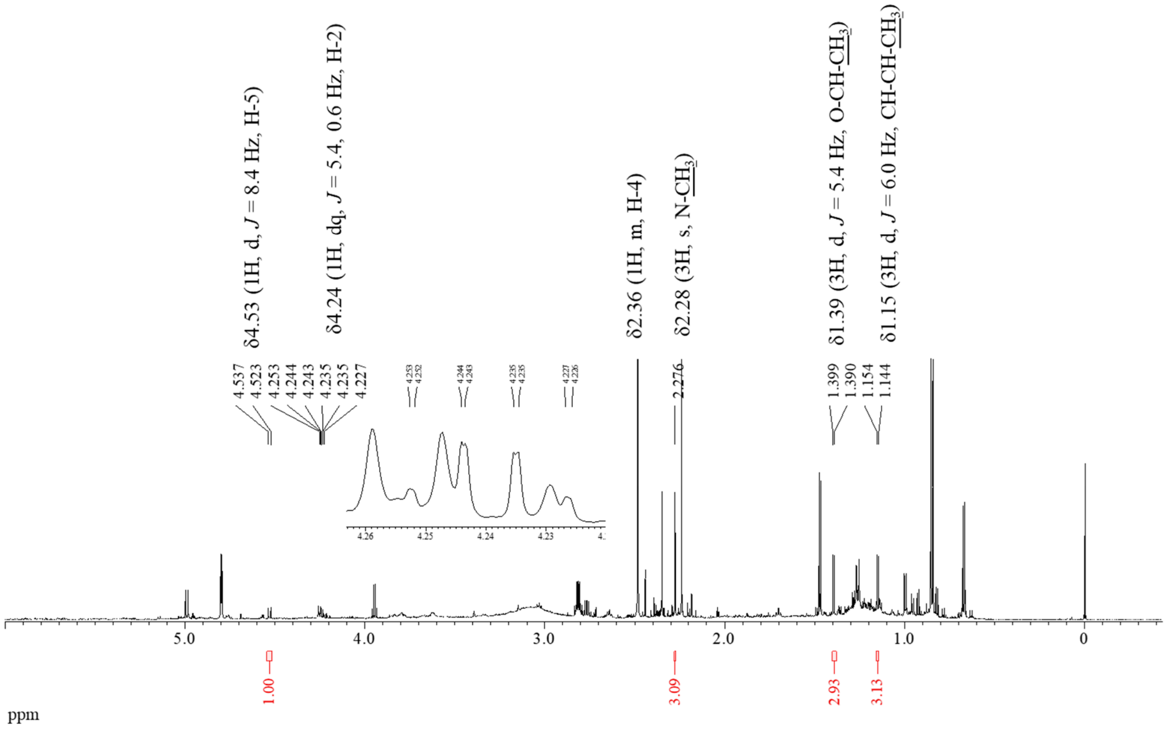

Figure S12. Characteritic carbon signals of compound **9**.

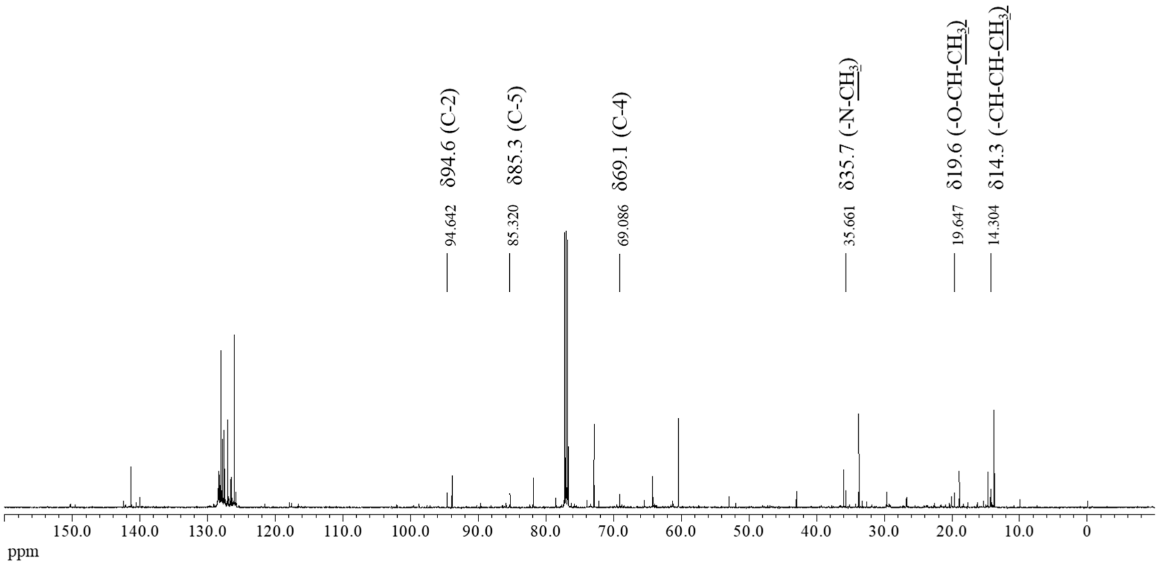

Figure S13. HMBC of compound **9**.

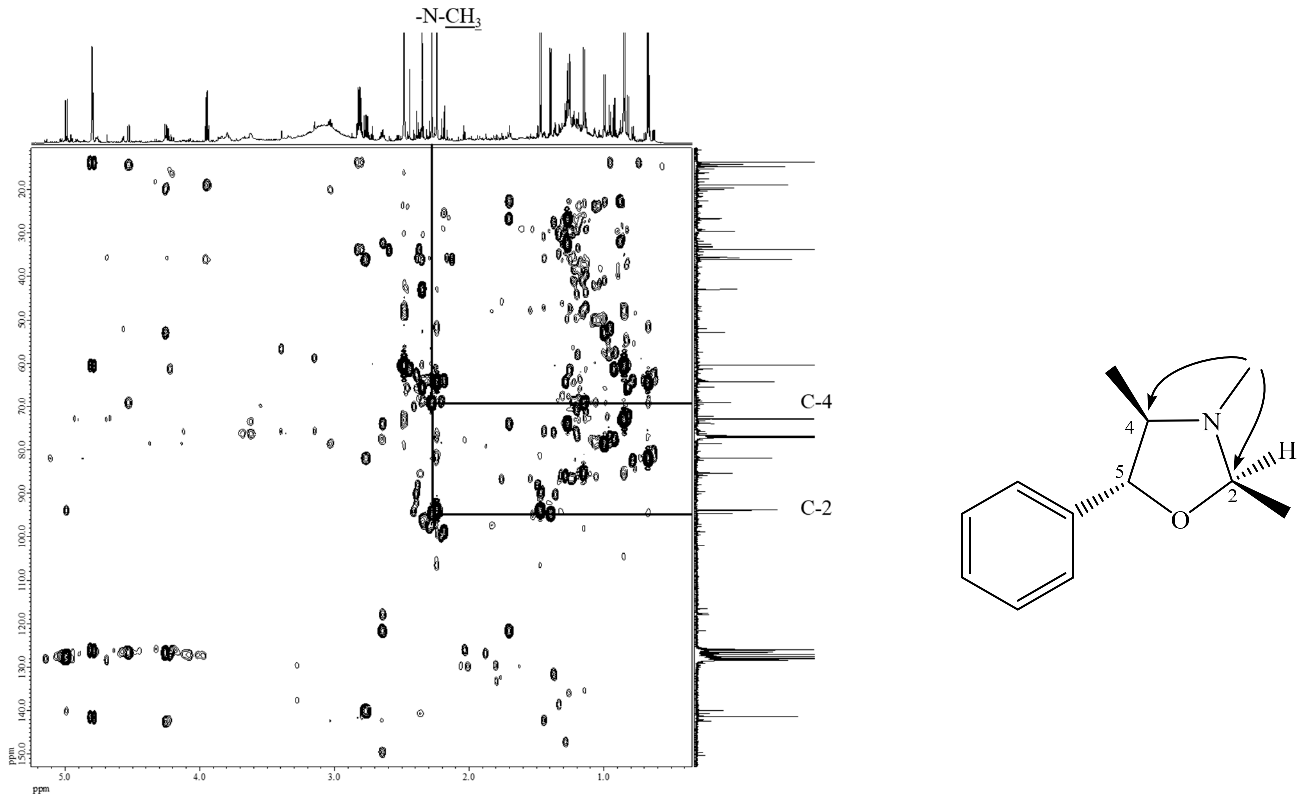

Figure S14. The ephedrine alkaloids chloroform extracts of *E. sinica* (EP01).

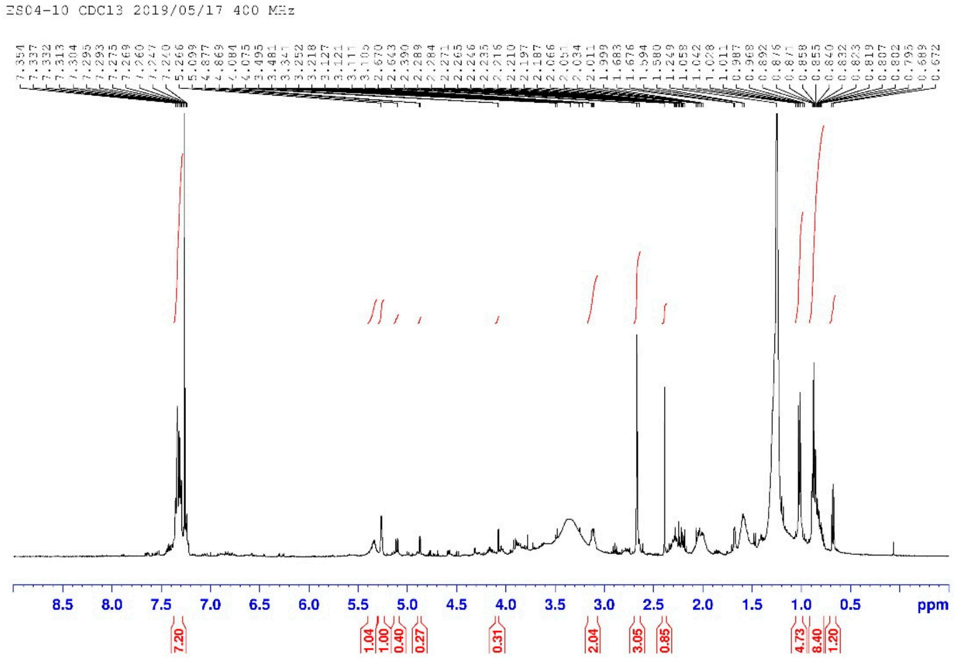

Figure S15. The ephedrine alkaloids benzene extracts of *E. sinica* (EP04).

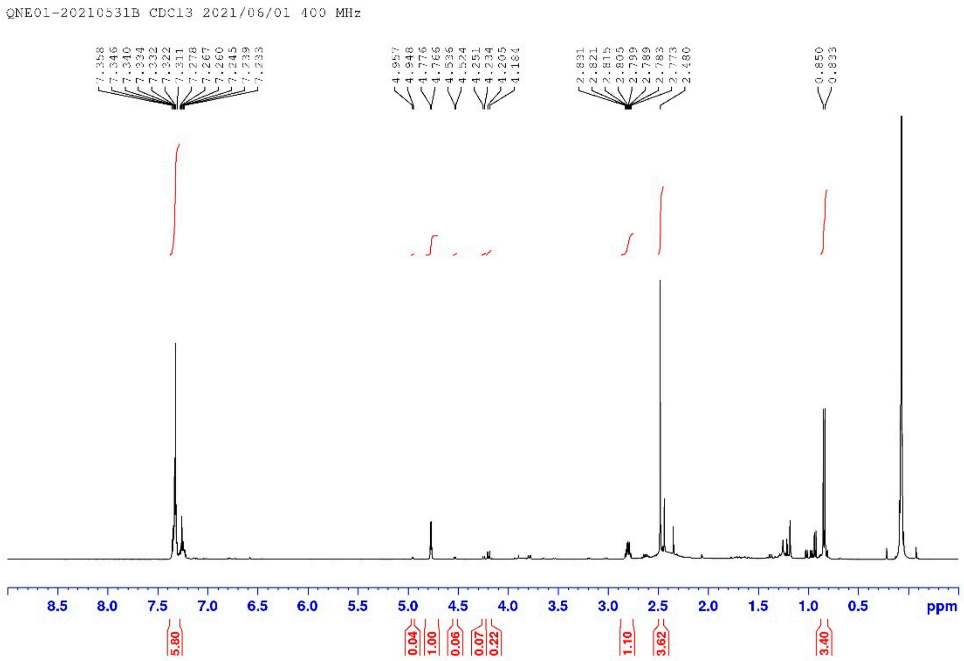

Figure S16. ^{1}H-NMR of ME (**1**).

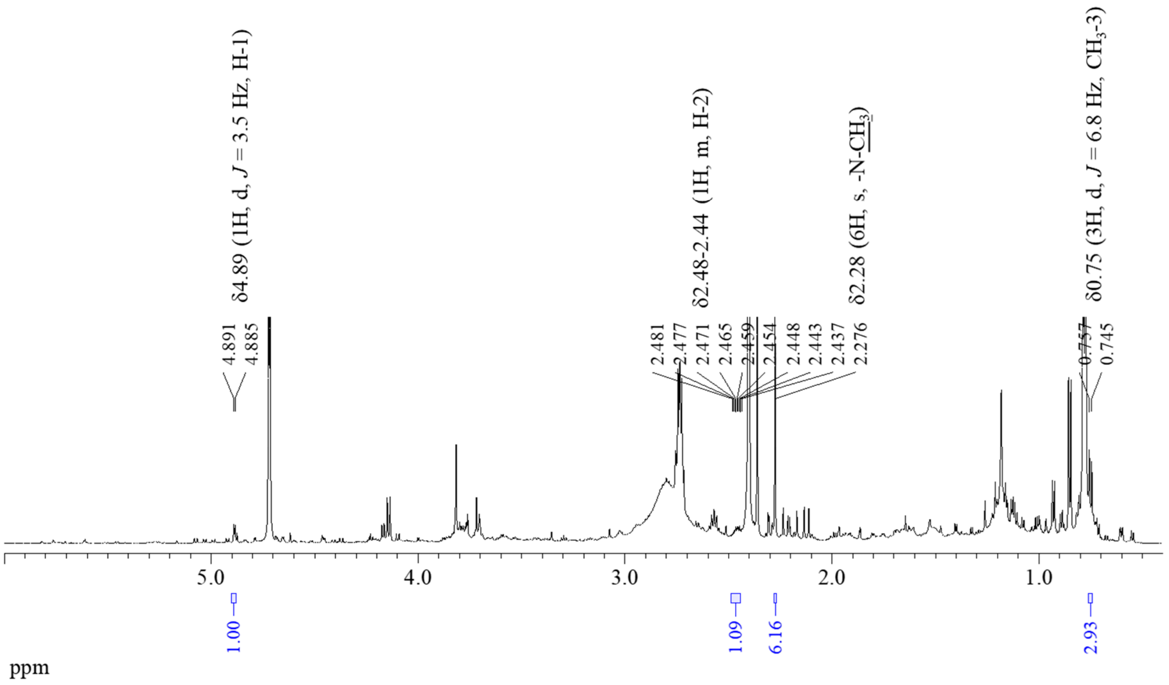

Figure S17. HSQC/TOCSY (▬▬), HSQC (▪▪▪▪▪) and HMBC (▬ ▬) spectra of ME (**1**).

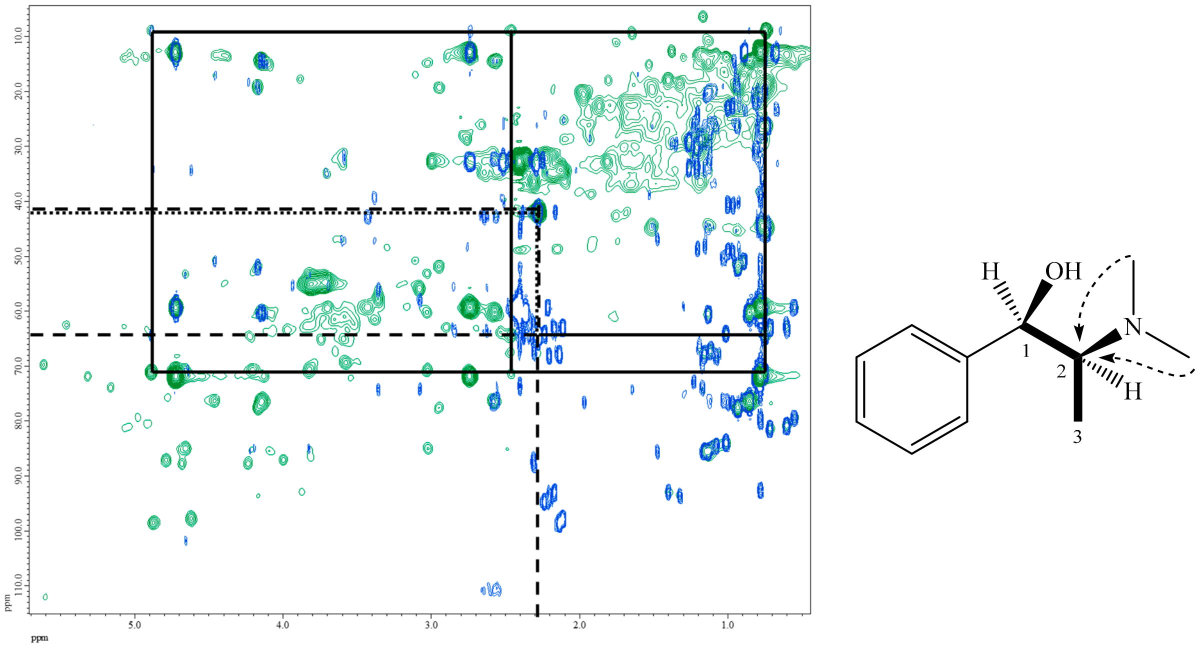

Figure S18. ^{1}H-NMR of EP (**2**).

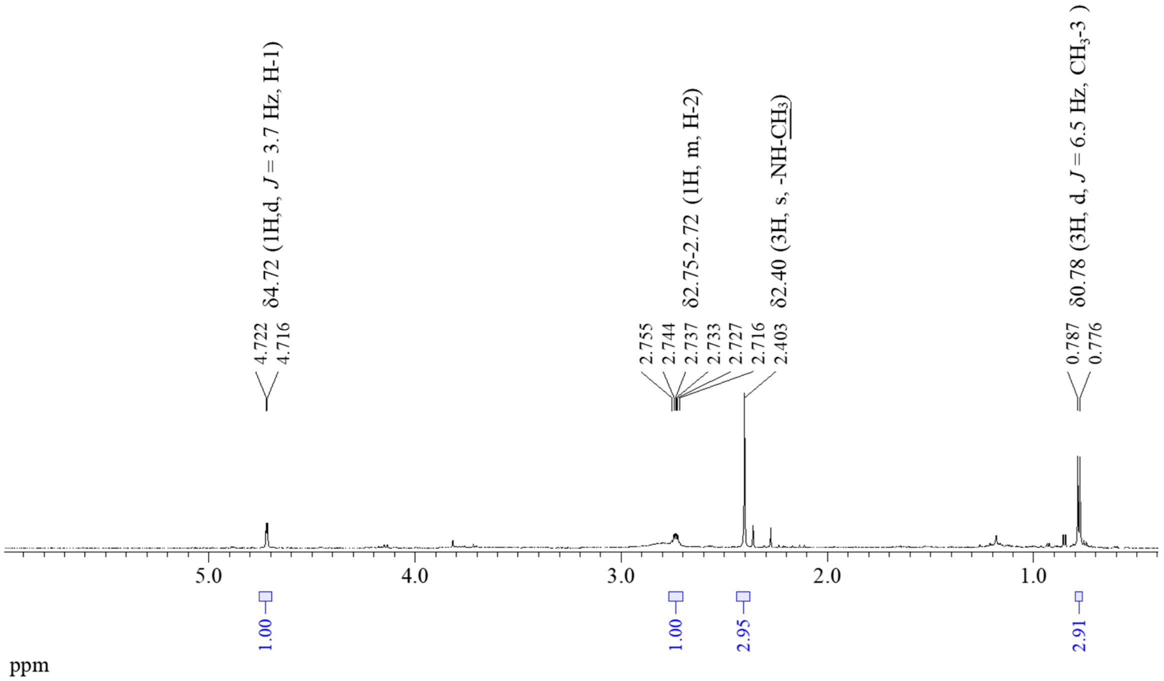

Figure S19. HSQC/TOCSY (▬▬), HSQC (▪▪▪▪▪) and HMBC(▬ ▬) spectra of EP (**2**).

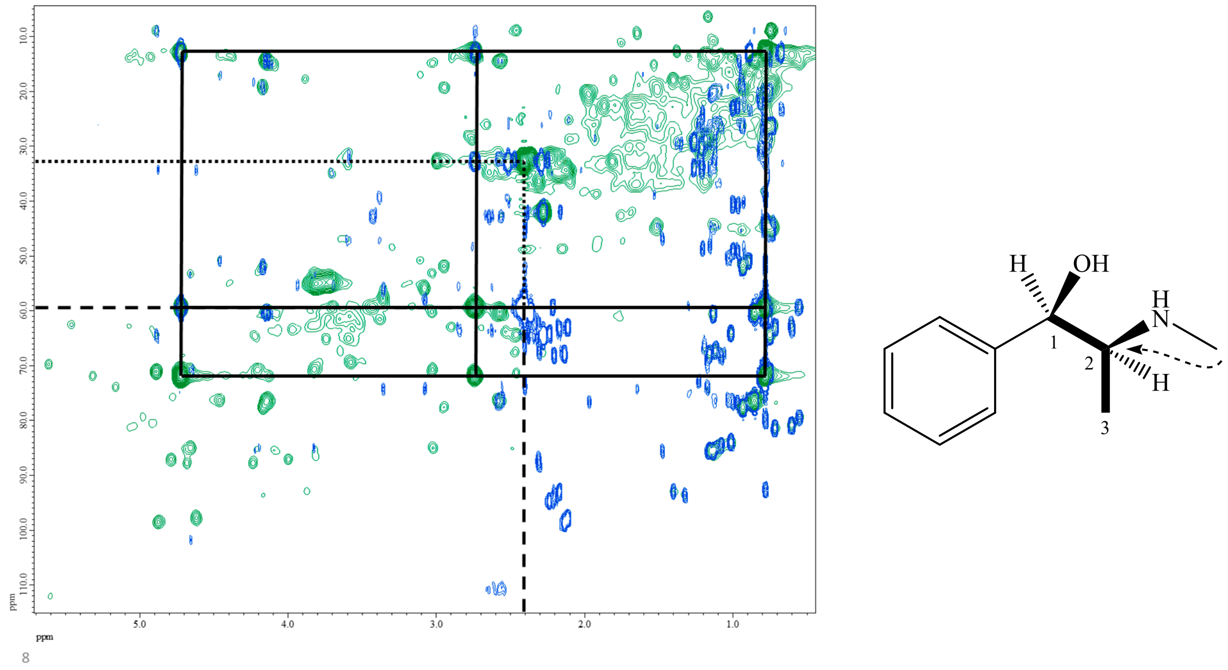

Figure S20. ^{1}H-NMR of NP (**4**).

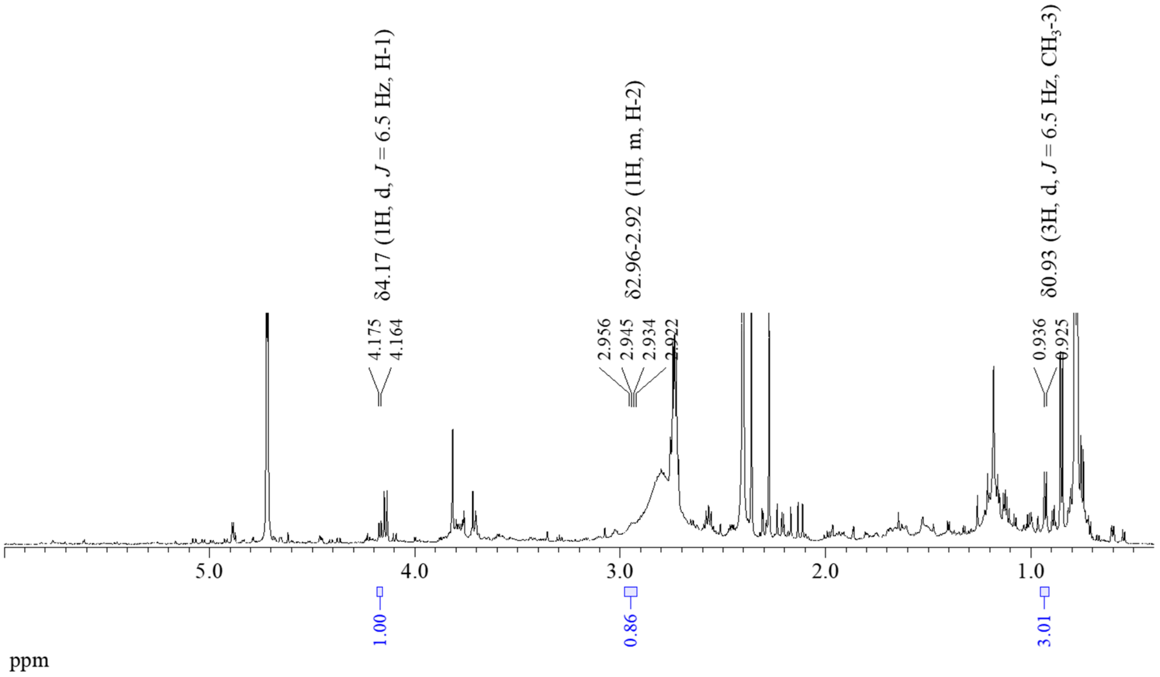

Figure S21. HSQC/TOCSY (▬) spectra of NP (**4**).

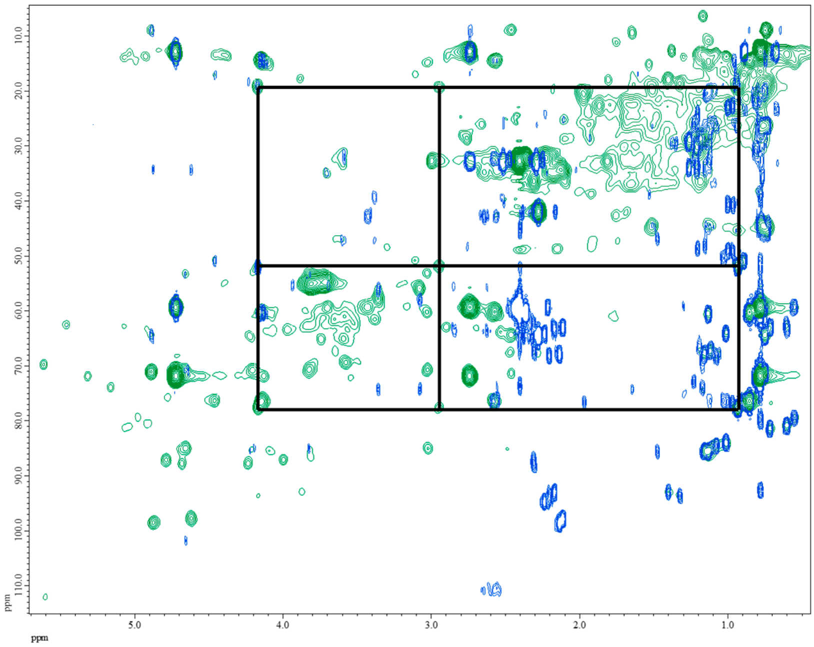

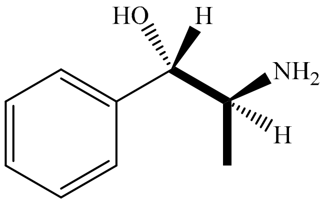

Figure S22. ^{1}H-NMR of PE (**6**).

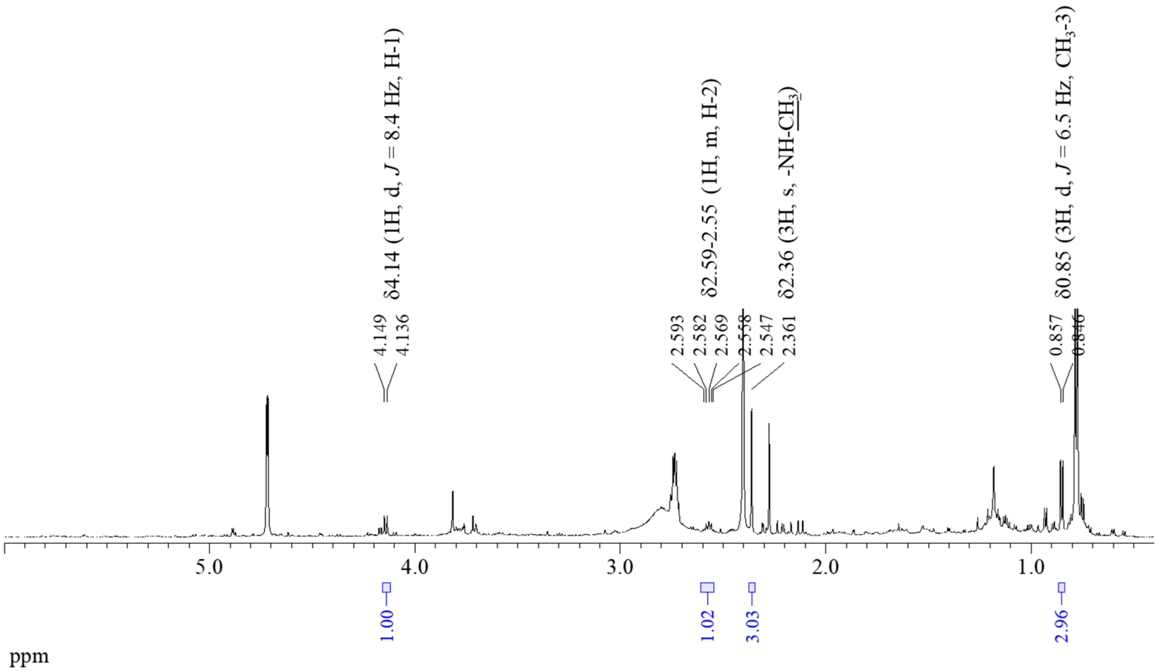

Figure S23. HSQC/TOCSY (▬▬), HSQC (▪▪▪▪▪) and HMBC(▬ ▬) spectra of PE (**6**).

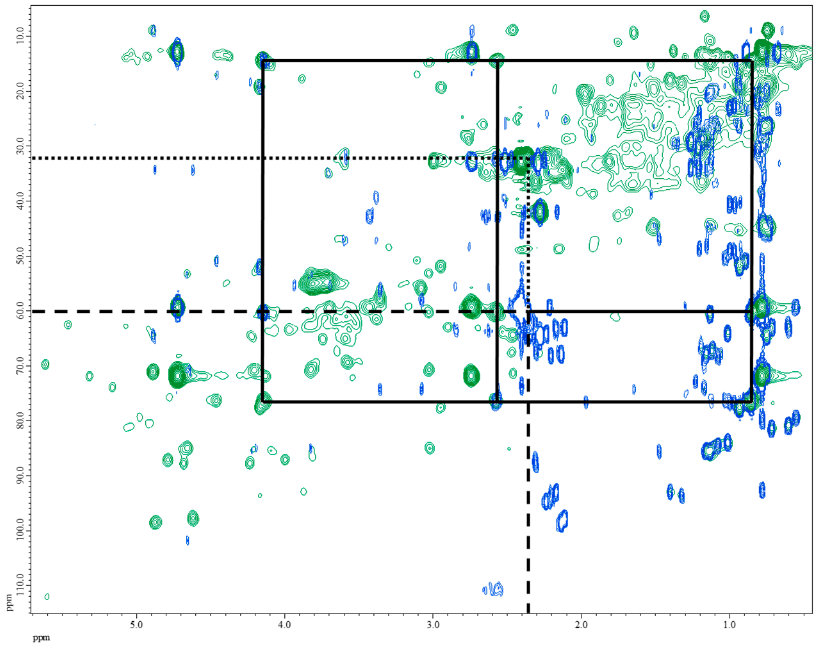

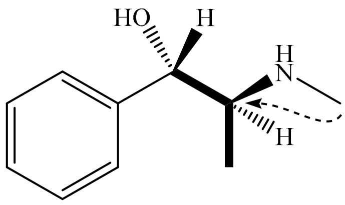

Figure S24. ^{1}H-NMR of EP01.

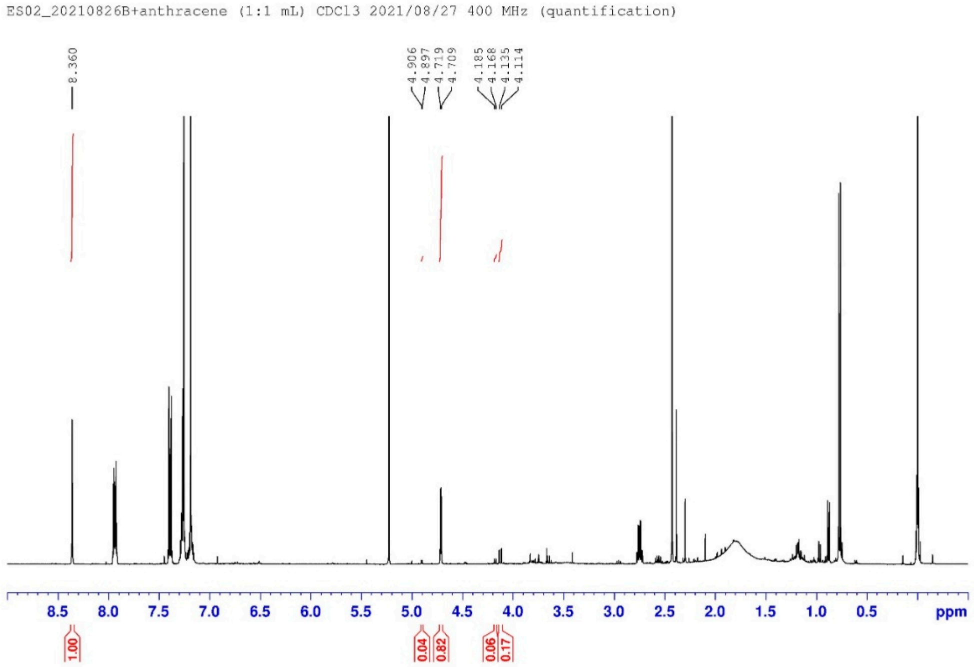

Figure S25. ^{1}H-NMR of EP02.

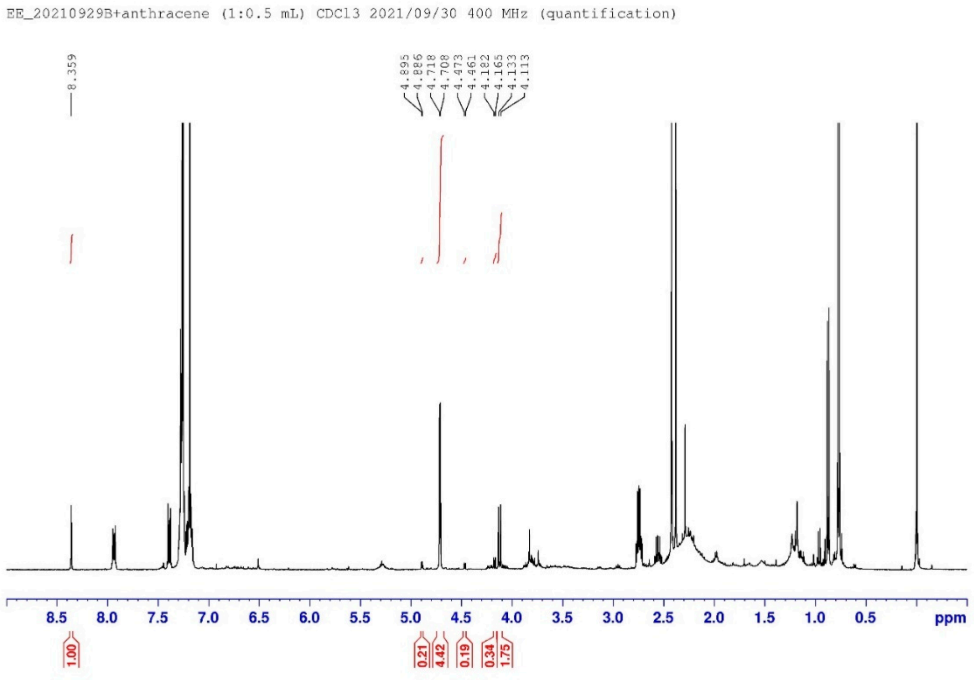

Figure S26. ^{1}H-NMR of EP03.

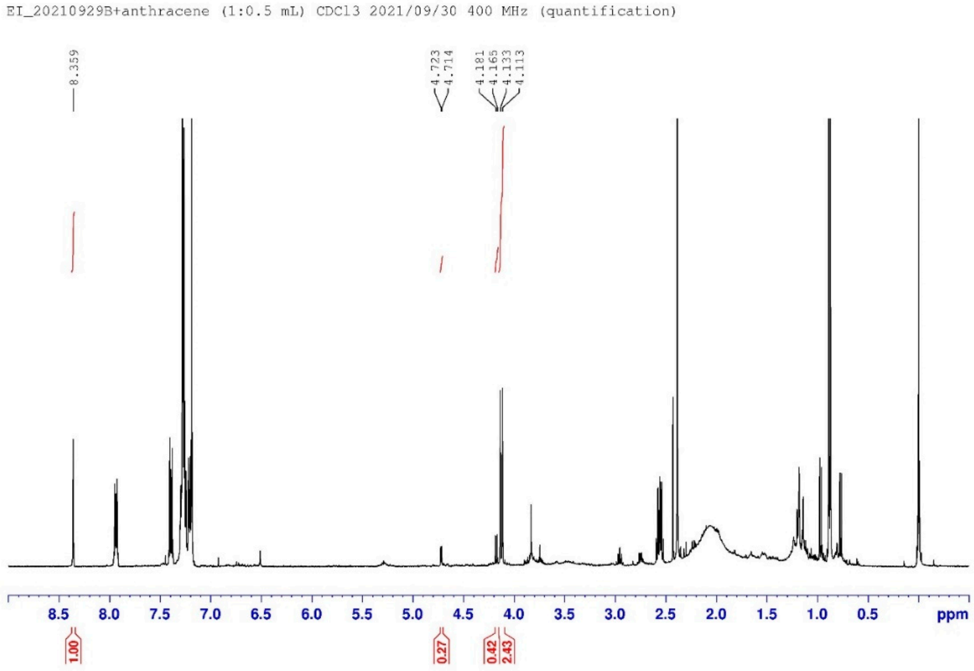

Figure S27. ^{1}H-NMR of EP04.

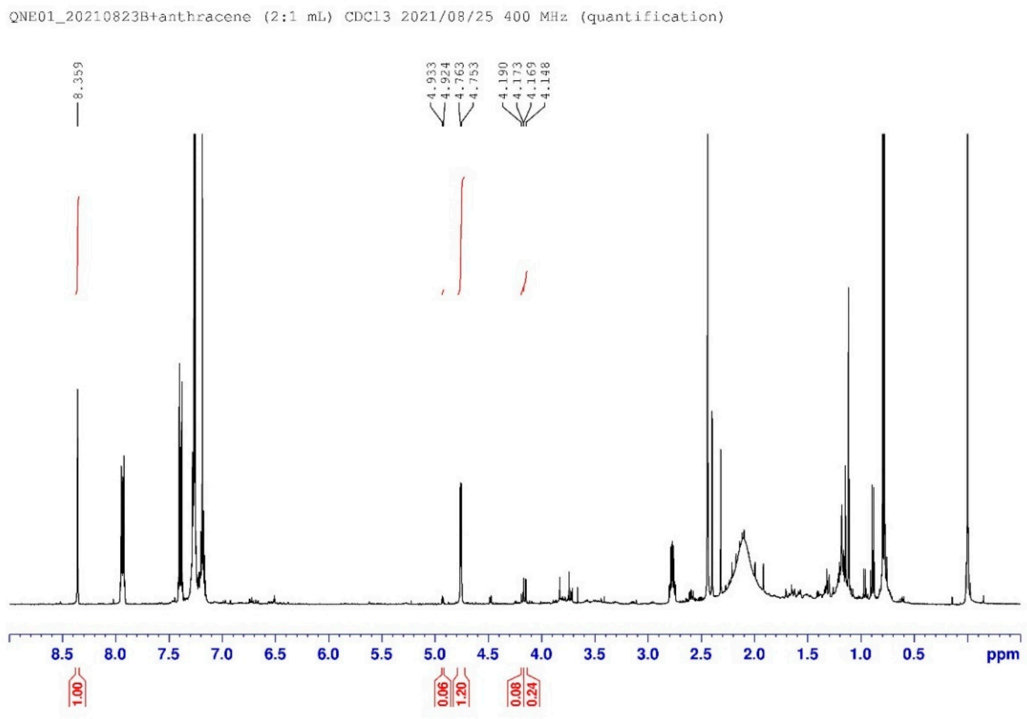

Figure S28. ^{1}H-NMR of EP05.

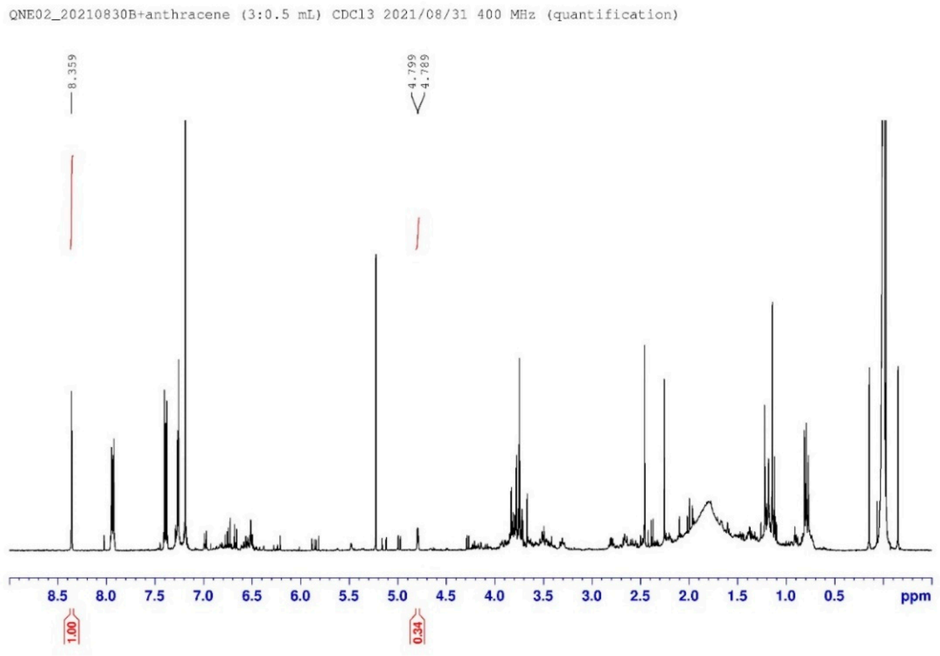

Figure S29. ^{1}H-NMR of EP06.

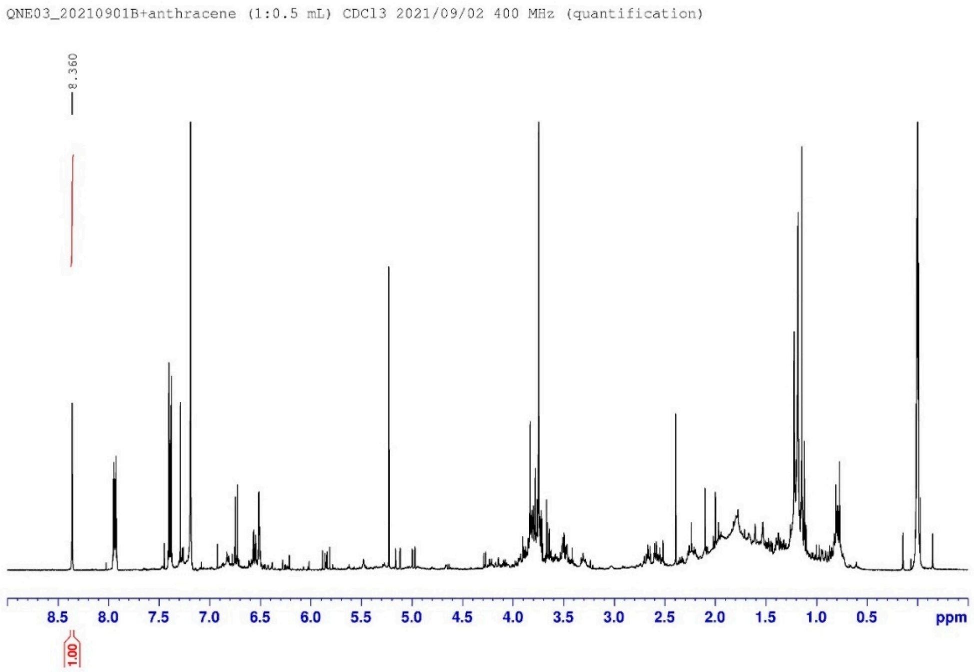

Figure S30. ^{1}H-NMR of EP07.

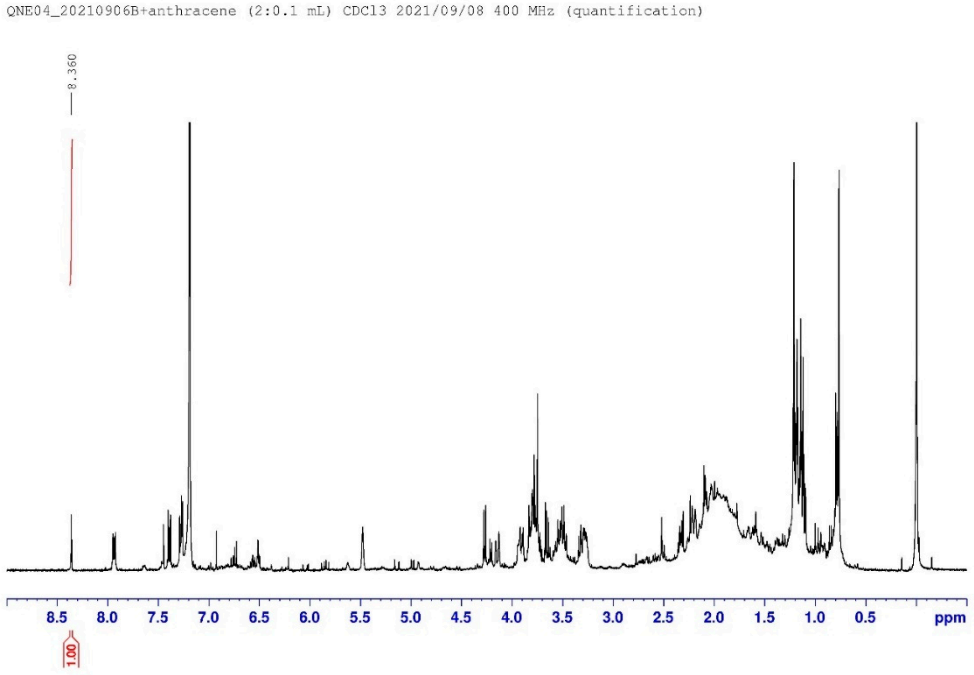

Figure S31. ^{1}H-NMR of EP08.

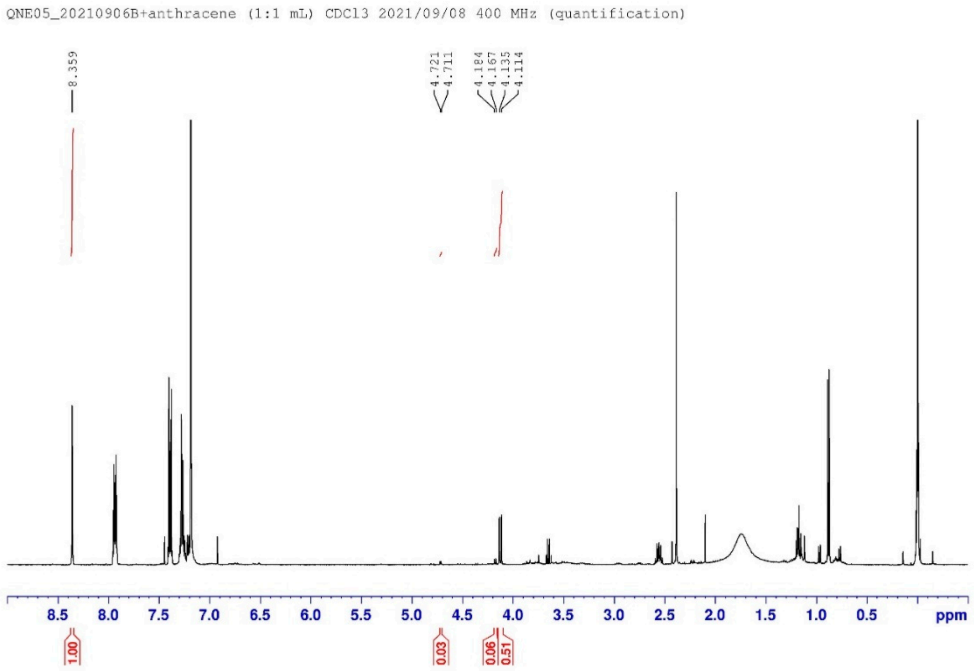

Figure S32. ^{1}H-NMR of EP09.

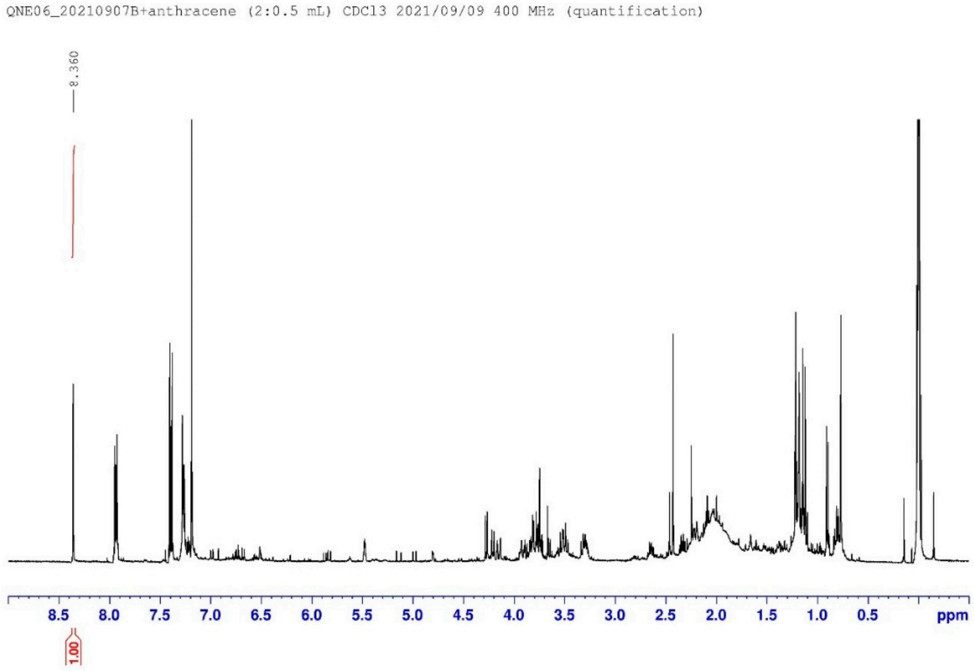

Figure S33. ^{1}H-NMR of EP10.

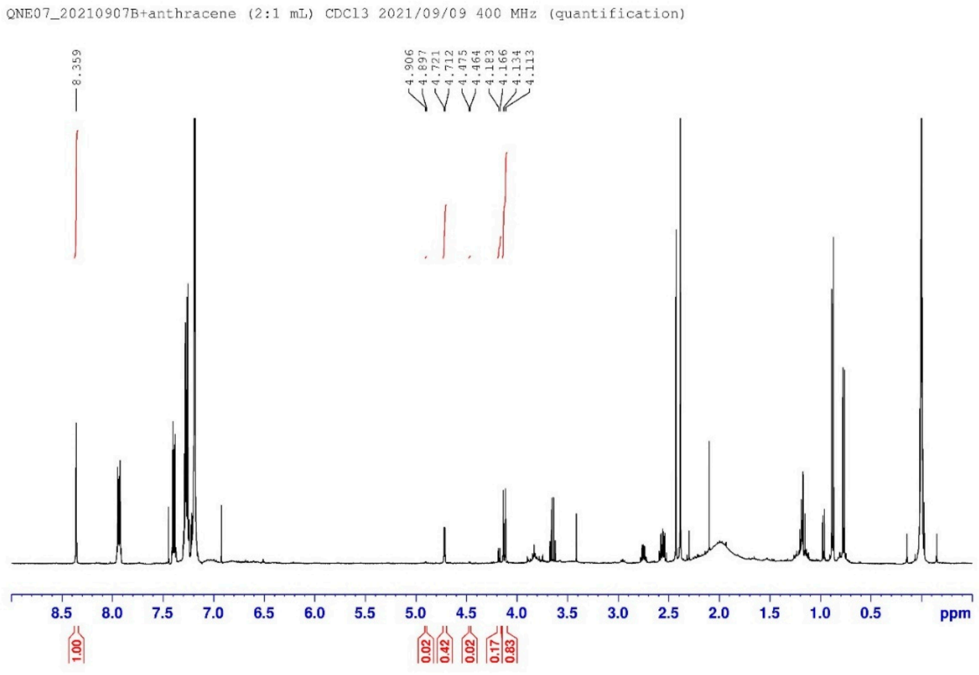

Figure S34. ^{1}H-NMR of EP11

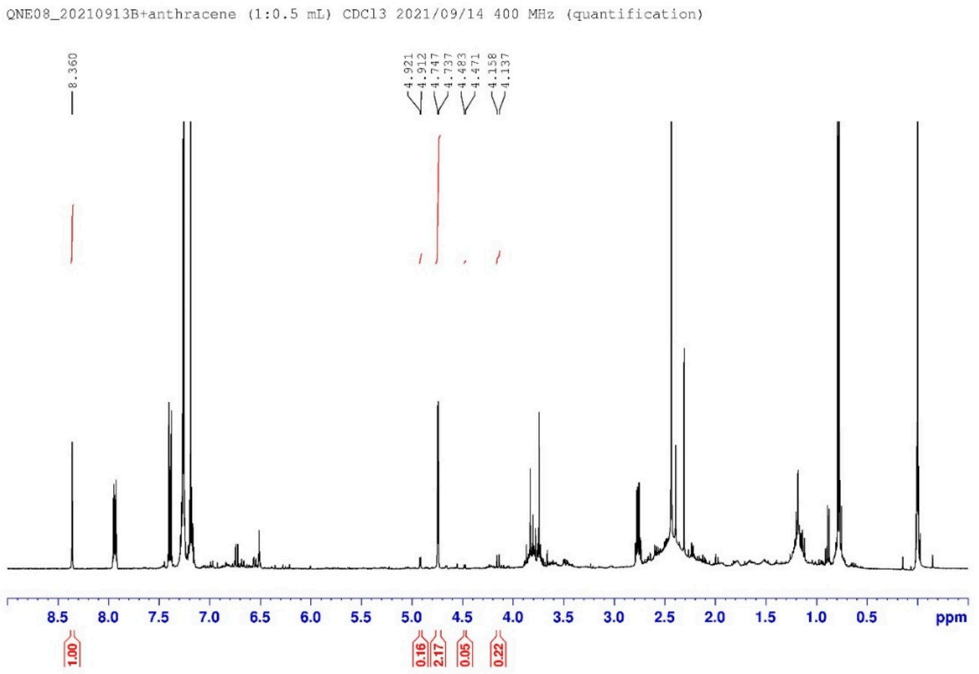

Figure S35. ^{1}H-NMR of EP12.

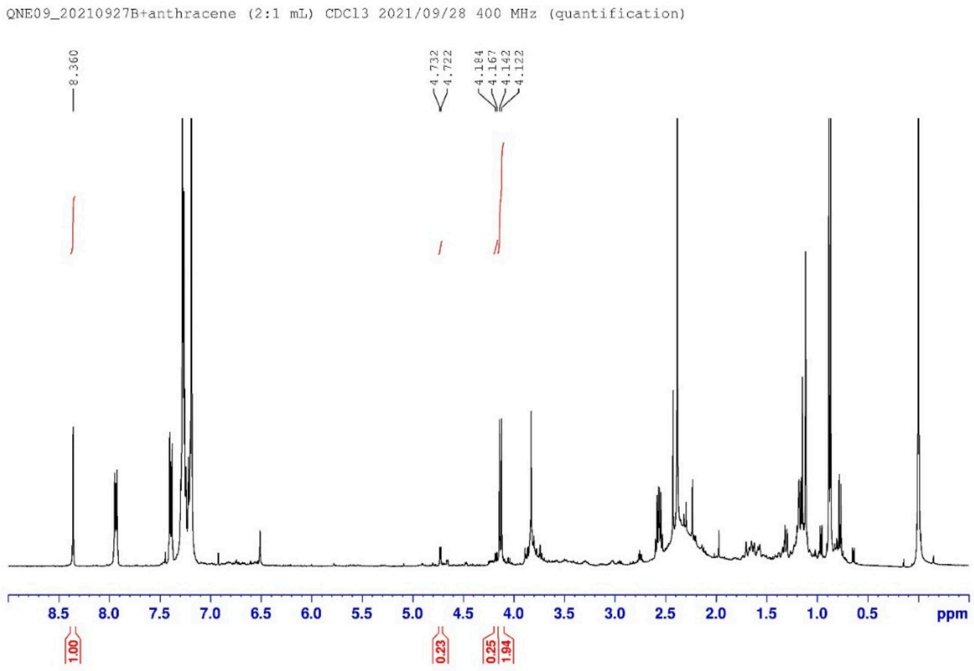

Figure S36. ^{1}H-NMR of EP13.

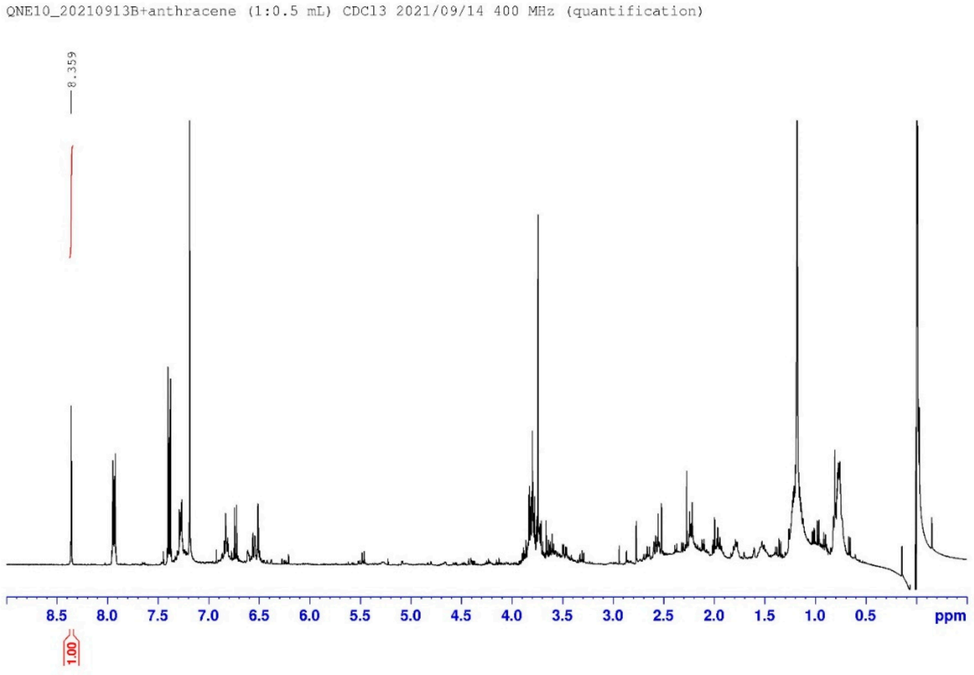

Figure S37. ^{1}H-NMR of EP14.

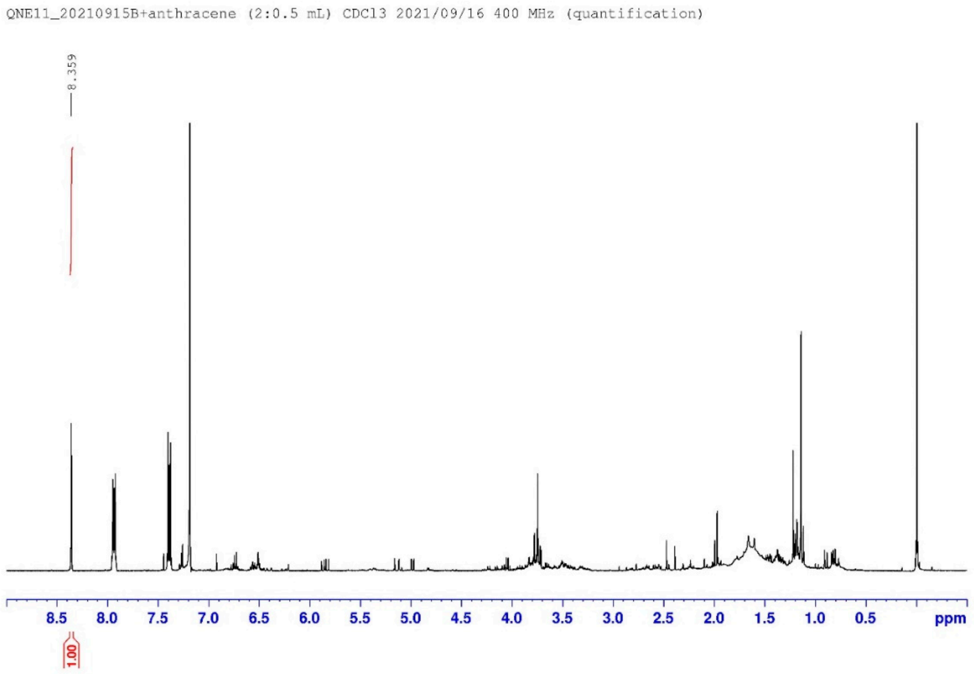

Figure S38. ^{1}H-NMR of EP15.

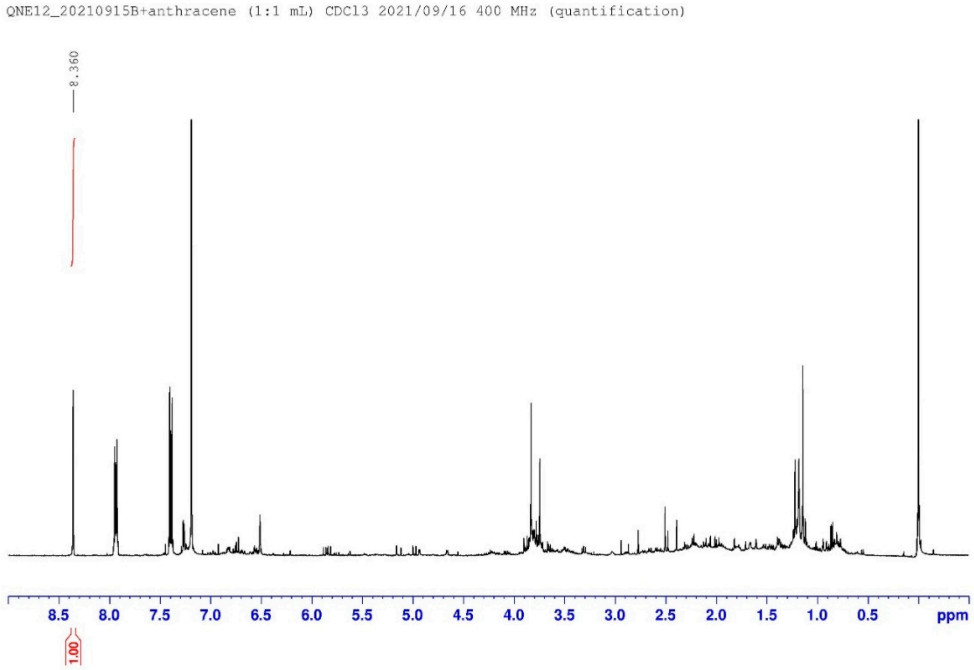

Figure S39. ^{1}H-NMR of EP16.

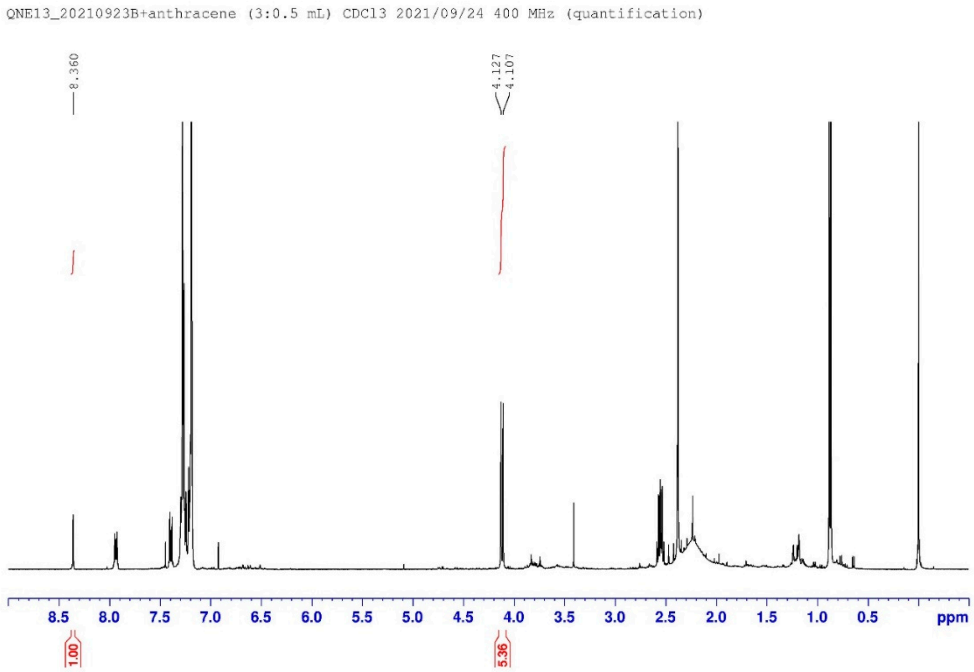

Figure S40. ^{1}H-NMR of EP17.

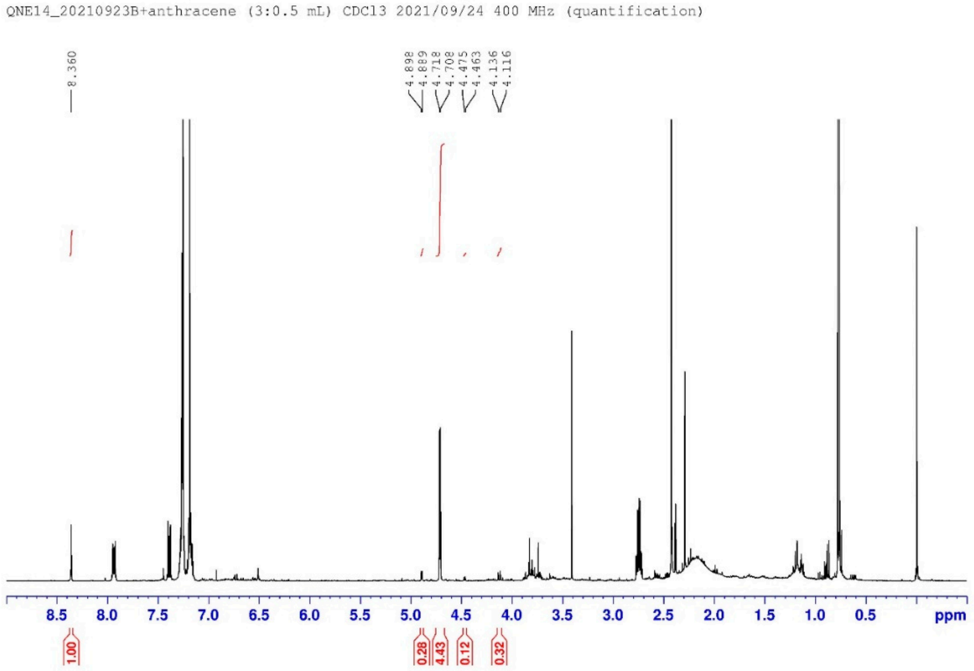

Figure S41. ^{1}H-NMR of EP18.

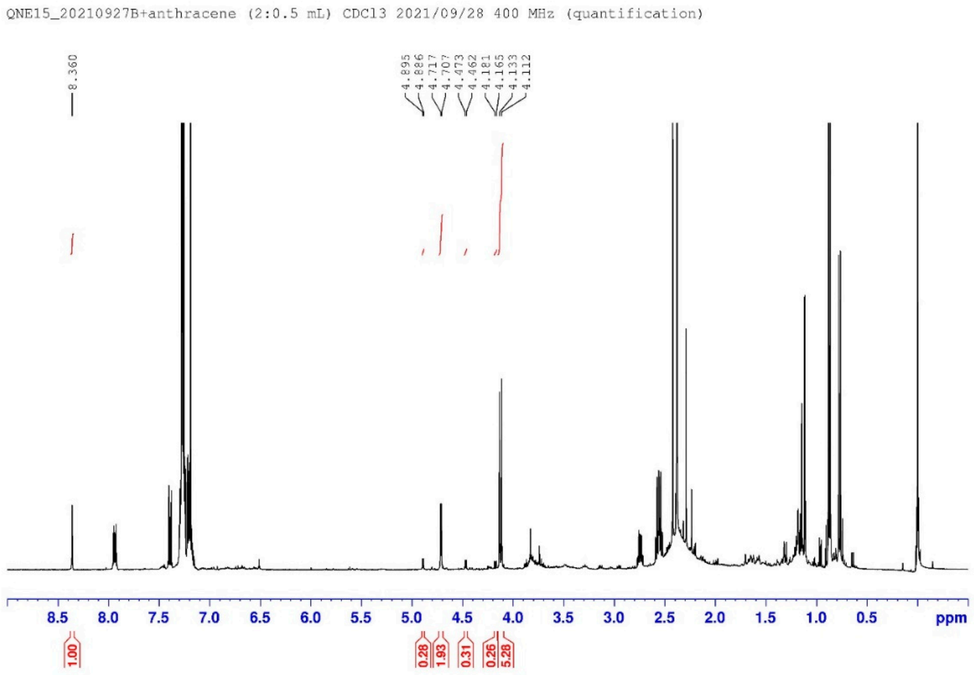

Figure S42. ^{1}H-NMR of EP19.

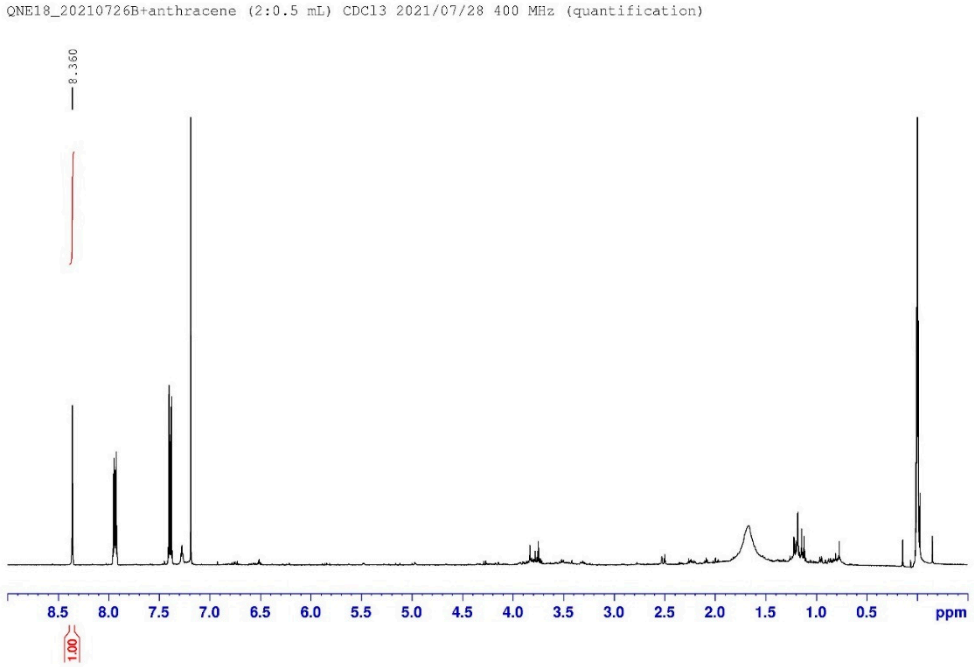

Figure S43. ^{1}H-NMR of EP20.

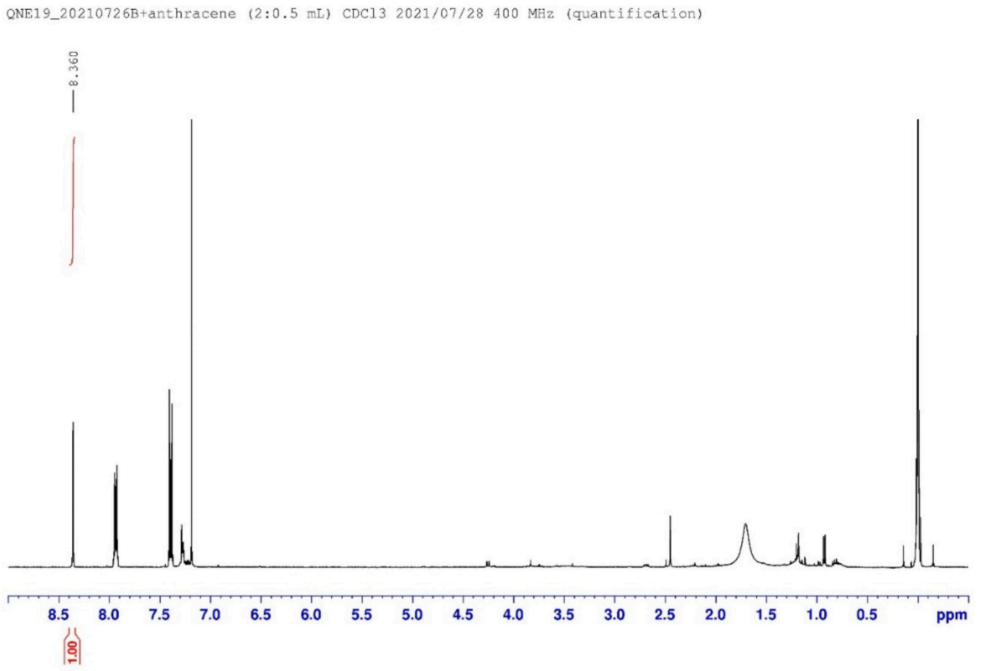

Table S1. Extraction and preparation of Ephedrae Herba for NMR analysis.

	**Sample**	**Source**	**Powder (g)**	**Extract (mg)**	**Anthracene Standard (mg/10mL)**
1	EP01	*E. sinica*	2.0395	27.47	5.92
2	EP02	*E. equisetina*	2.1106	12.07	5.16
3	EP03	*E. intermedia*	2.0593	12.48	5.16
4	EP04	*E. sinica*	2.0033	19.60	5.92
5	EP05	*E. sinica*	2.003	31.45	6.26
6	EP06	*E. intermedia*	2.0176	8.80	6.26
7	EP07	*E. przewalskii*	2.0051	5.91	5.49
8	EP08	*E. glauca*	2.0268	19.74	5.49
9	EP09	*E. przewalskii*	2.0003	9.93	5.49
10	EP10	*E. equisetina*	2.0118	22.39	5.49
11	EP11	*E. gerardiana*	2.0508	9.64	8.11
12	EP12	*E. intermedia*	2.239	12.07	5.62
13	EP13	*E. lepidosperma*	2.0272	12.75	8.11
14	EP14	*E. minuta*	2.068	6.27	8.11
15	EP15	*E. regeliana*	2.0518	3.65	8.11
16	EP16	*E. monosperma*	2.0465	13.65	5.62
17	EP17	*E. saxatilis*	2.0928	15.66	5.62
18	EP18	*E. sinica*	2.2834	15.54	5.62
19	EP19	*E. przewalskii*	1.7584	13.40	10.96
20	EP20	*E. intermedia*	0.8773	11.37	10.96
